# Supplementary material for: Patterns of Intron Gain and Loss in Fungi
Source: PLoS Biol. 2004 Nov 30;2(12):e422. doi: 10.1371/journal.pbio.0020422 (PMC532390; doi:10.1371/journal.pbio.0020422)
Supplement: Table S1 — Also available at http://genes.mit.edu/NielsenEtAl/. (4.3 MB ZIP). [file pbio.0020422.st001.zip › NielsenEtAl/html/724.html]

AN0321.1.NCU01772.1.MG06984.1.FG00843.1


```
 CLUSTAL W (1.82) Multiple Sequence Alignments - Introns Inserted


Sequence 1: NCU01772.1	1155 aa
Sequence 2: FG00843.1	1157 aa
Sequence 3: MG06984.1	1158 aa
Sequence 4: AN0321.1	1656 aa
Alignment Length: 1671 aa
Number Identitical Residues: 642 aa
Alignment Score (without introns) 30810


MG06984.1 	------------------------------------------------------------
NCU01772.1	------------------------------------------------------------
FG00843.1 	------------------------------------------------------------
AN0321.1  	MDTGIIGPVTTMDSFVEEFGNRSATVHGLLVSSILIPAAISSFFAGYLADKFGRPQGISI
          	 .:.  .. :: .:  .. .. :::  .   ::   .:: ::  :.  :.. . ... : 

MG06984.1 	-----------------------------------------~------------------
NCU01772.1	-----------------------------------------~------------------
FG00843.1 	-----------------------------------------~------------------
AN0321.1  	GTLIFGLGAALEAGAVHVSMFVLGRCIEGMGEGLYLGTLVV2YICEISPPGVRGALTTGP
          	.:   . .:: .:.:   :    . . .. ...   .:      .. :...  .: ::..

MG06984.1 	------------------------------------------------------------
NCU01772.1	------------------------------------------------------------
FG00843.1 	------------------------------------------------------------
AN0321.1  	QLLITLGLVVGFFTCYGTARIQSSMSWRTPFLILACLSLLFSAAAFLWLVPSPRWLTIHG
          	.   : .   .  :. .::  .:: :  :.    :. :   ::::     .:.   :  .

MG06984.1 	------------------------------------------------------------
NCU01772.1	------------------------------------------------------------
FG00843.1 	------------------------------------------------------------
AN0321.1  	RREEANVVWDLLGVSHAEREKSEIEQARGSVIQGPVSQEVAVTNVSSMDLTVATPQVEDK
          	  ..:.   .  . : :. ..:. ..: .:  ... :.. : :. :: . : ::.. ...

MG06984.1 	--------------------------------------~---------------------
NCU01772.1	--------------------------------------~---------------------
FG00843.1 	--------------------------------------~---------------------
AN0321.1  	SIRHKLFDIFSKDVRSRTALAVFLMAMQQLSGIDGVLY0YAPLLFEQAGLASADASFFAS
          	:   .  .  :..  : :: :    : .. :. ..     :.   ..:. :::.::  ::

MG06984.1 	------------------------------------------------------------
NCU01772.1	------------------------------------------------------------
FG00843.1 	------------------------------------------------------------
AN0321.1  	GVSALVIFAVTIPALIWADKWGRRHSVIYGGVVISLTMFLMGALYAGNAVHSSAGAGRWV
          	. ::    : : .:   :.. .   :   ..   : :    .:  :..:  :::.:.   

MG06984.1 	------------------------------------------------------------
NCU01772.1	------------------------------------------------------------
FG00843.1 	------------------------------------------------------------
AN0321.1  	VIVSIYIFAVIFSLSWAVGIKIYAAEIQPQRTRASATSLAHGSNWATNFLVALTTPILLD
          	   :    :   : : : . .  ::. ... : ::::: : .:. ::.   : ::.   .

MG06984.1 	-------------~-------------------~--------------------------
NCU01772.1	-------------~-------------------~--------------------------
FG00843.1 	-------------~-------------------~--------------------------
AN0321.1  	KSSFGAYFLFGGC1MPPRKRAVAPASARAVQSE0TTGSTKSTTETARRKRAAQAKEAKEA
          	.:: .:    ...  .. . : :.::: : .:. ::.::.:::.::  . ::.:..:..:

MG06984.1 	--------------------------MPSVVDDGFEALLEPFYNGKKLTDPISTAQDKWQ
NCU01772.1	---------------------------MAALDDGFEALLEPFYNGKKLTDPISTTQDKFH
FG00843.1 	---------------------------MPTVDDGFEALLEPFYNGKKLTDPISTKEDKFQ
AN0321.1  	TTSEHPTQGEKDNVADAETEPVPEPKYPYRHRDPFDALLEPFYYNKSLTDPISTAKDKWN
          	:::. .:...... :.:.:.. ....      * *:******* .*.******* :**::

MG06984.1 	LLPAFLKVKG1LVKQ2HIDSFNFFINTEIKEIVKANRIVRSDVESGFFLE~FTDIRVGKP
NCU01772.1	LVPAFLKVKG~LVKQ~HIDSYNYFVNEEIKDIVRANRVVRSDQDPNFWLE~FRDIRVGMP
FG00843.1 	LLPAFLKVKG1LVKQ2HIDSYNFFVEHEIKDIVRANRTIRSEVDSNFWLE2FTDIRVDKP
AN0321.1  	LLPAFLKVKG~LVKQ~HIDSYNYLVEVQLKKIVESSNTIRSDVDHNFYIK~FTDIYLGFP
          	*:******** **** ****:*:::: ::*.**.:.. :**: : .*::: * ** :. *

MG06984.1 	NR-AEYSDIK---AQNEVSPMECRLRDMTYAAPIKVDIAYFRDKKKVLRKNVTLGRMPIM
NCU01772.1	TRTEDGEQVQ---SRNPVTPMECRLRDLTYAAPVEVDIAYTRDKNVIVRKNIPLCRIPVM
FG00843.1 	RR-QDWQDAK---SHTEVTPMECRLRDMTYAAPISVDIQYIRDKQRIVRKNVPLGRMPVM
AN0321.1  	RRADEPQDVRTEFEESTVTPHECRLRDTTYAAPIQVDFEYIRGRQRVRRKGVSIGRMPVM
          	 *: : .: ::.  .. *:* ****** *****:.**: * *.:: : **.:.: *:*:*

MG06984.1 	LKSSNCWLSGANNAEMEDMNECPLDPGGYFIINGTEKVILIQEQLSKNRVIVEADEKNNV
NCU01772.1	LKSAKCYLNGATNAQMEVFNECPLDPGGYFVIGGTEKVILIQEQLSKNRIIVEADEKTGG
FG00843.1 	LKSSICRLGGANNTKMEEMNECPLDPGGYFIIGGTEKVILIQEQLSKNRIIVEADEKNNI
AN0321.1  	LRSSKCVLGNKTPAQMTVLNECPLDPGGYFIVNGTEKVILVQEQLSKNRIIVETDPKKEI
          	*:*: * *.. . ::*  :***********::.*******:********:***:* *.  

MG06984.1 	ISASVTS~STHERKSKTYVTLKKERITLAHNTLVEPIPIAIILKALGGLPDHEIIQLVAG
NCU01772.1	VMASVTS~STHERKSKTYVVLKKDRIVLQHNTLSEPIPIVIVLKAFGGLSDNDIIELVCG
FG00843.1 	ISASVTS~STHERKSKTYVTLKKDRILLTHNVLVEGIPIVIILKALGGLSDMEIMQLVAG
AN0321.1  	VQASVTS2SSNERKSKSYIILKKDRLYVKHNVLSEDIPIVILLKAMGIHTDKEMMLLVAG
          	: ***** *::*****:*: ***:*: : **.* * ***.*:***:*  .* ::: **.*

MG06984.1 	EDARYQDDFAVNFDDAARAGVFTQQQSLEYIGSRVKMGSNRFKTPGGQAPRRNNAEEGLE
NCU01772.1	GDARYQDDFLINFDEAGRAGVYTQQQALEYVGARVKMGGNRPK--FGAAPRRNHAEEGLD
FG00843.1 	SDGRYQDEFLVNFDEATKVGVFTQHQALEYIGTRVKMAPRRGI--FGPQVRRNHVEEGLD
AN0321.1  	VDKVYQEDFAINFEEAIKVGVFTQQQALDWIGSRIKINRKQMS------YRRTHVQEAVE
          	 *  **::* :**::* :.**:**:*:*:::*:*:*:  .:         **.:.:*.::

MG06984.1 	ALANMIIGHVPILDLDFYPKACYVAMMVRRVLMAAHDHKLVDDRDFVGNKRLELAGQMLS
NCU01772.1	ALANLVIAHVTVEGLDFYPKAVYIAMMVRRVLMAAQNPKLVDDRDFVGNKRLELAGQLIS
FG00843.1 	ALANLVIAHVPIEGLDFYPKAIYVAMMTRRVLIAAHNPKLVDDRDFVGNKRLELAGQLLS
AN0321.1  	AIASVIISHIEVKDMNFRPKAIYVANMARRVLMAKNDAALVDDRDYLGNKRLELAGQLLA
          	*:*.::*.*: : .::* *** *:* *.****:* ::  ******::**********:::

MG06984.1 	LLFEDLFKKFLVDLRFNIDKVLRKSNRGVAFDPLNSITATSGHITAGMNRAISSGNWTVK
NCU01772.1	LLFEDLFKKFISDVKYNIDNFFKKPNRTSTYDPHGAIMANGHYISHGLNRAIQSGNWTVK
FG00843.1 	LLFEDLFKQFTTNVRMSIDKFLKKNNRAVPLDAVNMISNHANNIGYGINRAIQTGNWNVK
AN0321.1  	LLFEDLFKKFCFDIKMNIDKVLNKRNRVEAFDAWSVIGMHSNHITQGMNRAISTGNWSLK
          	********:*  ::: .**:.:.* **  . *. . *   .  *  *:****.:***.:*

MG06984.1 	RFNMNRAGVTHVLSRLSYISALGMMTRISSQFEKTRKVSGPRALQPSQWGMLCTSDTPEG
NCU01772.1	RFNMNRAGITHVLSRLSYIAALGMMTRISSQFEKTRKVSGPRALQPSQWGMLCTSDTPEG
FG00843.1 	RFNMNRAGVTHVLSRLSYIAALGMMTRISSQFEKTRKVSGPRALQPSQWGMLCTSDTPEG
AN0321.1  	RFRMERAGVTHVLSRLSYIAALGMMTRISSQFEKTRKVSGPRALQPSQFGMICLADTPEG
          	**.*:***:**********:****************************:**:* :*****

MG06984.1 	EACGLVKNLALMTHITTHFEEDPVKEWILAMDSGAEALSNFSGSEMHTPGTFVITVNGTP
NCU01772.1	EACGLVKNLALMTHITTNVPEEPVKKMIFAADESVGPIREYTGTEMHSKGSYIIHLNGTP
FG00843.1 	EACGLVKNLALMTHITTNVDEEPVKRWIFTLDAGVEPIRNFSGAEMHREGSYIIHVNGTP
AN0321.1  	EACGLVKNLALMTHITTNDEEGPIKNLIFMLG--AEDIQTVGGKEIYAPGSYTISINGTP
          	*****************:  * *:*. *:  .  .  :    * *::  *:: * :****

MG06984.1 	FALTWNPEEFCKKFRDTRRKGELSAFVSISINYHFATVNIATDEGRICRPYIIIENGVQR
NCU01772.1	FAVTREPKRFAARFRTLRRRGWVSPFVSIHINEHFSAVHIATDEGRICRPYIIVKNGKSM
FG00843.1 	FALTRYPKRFAQKFRTMRRRGWISPFVGININTHFNAVHIATDEGRICRPYIIVKNGKQK
AN0321.1  	MALTRRPKYFLNAFRRLRRMGRISEFVSIYINHHQRAVHVATDDGRICRPLIIVEDGKSR
          	:*:*  *: *   **  ** * :* **.* ** *  :*::***:****** **:::* . 

MG06984.1 	IKEEHLESLRQGDKDFDYFLNNGIVEYLDVNEEQDTLVALYEKDVTPAHTHLEIEPFTIL
NCU01772.1	LKAEHLKMLQLGQATFDTFLTSGVVEYLDVNEENDTLVALQEDQITNSTTHMEIEPFTIL
FG00843.1 	LKPEHLRLLQMGKATFDDFLNRGIVEYLDVNEENDALITIYEHQVTQSTTHLEIEPFTVL
AN0321.1  	VKKHHLRKLRDGTMQFDDFLAQGLVEYVDVNEENDSLIAIYEKDITDTTTHMEIEPFTIL
          	:* .**. *: *   ** **  *:***:*****:*:*::: *.::* : **:******:*

MG06984.1 	GAVAGLIPFPHHNQSPRNTYQCAMGKQAIGAIAYNQFNRIDTLLYTLVYPQRPMVITKTI
NCU01772.1	GAVAGLIPFPHHNQSPRNTYQCAMGKQAIGAIAYNQFNRIDTLLYTLVYPQRPMVITKTI
FG00843.1 	GAVAGLIPFPHHNQSPRNTYQCAMGKQAIGAIAYNQFNRIDTLLYTLVYPQRPMVISKTI
AN0321.1  	GAVAGLIPYPHHNQSPRNTYQCAMGKQAIGAIASNQFLRIDSILYLMVYPQKPMVKSRTI
          	********:************************ *** ***::** :****:*** ::**

MG06984.1 	ELIHYDKLPAGQNATVVVMSYSGYDIEDALVLNKASCDRGFGRCQVFRKHTAELQTYANN
NCU01772.1	QLIHYDKLPAGQNATVVVMSYSGYDIEDALVLNKASLDRGFGRCQVFRKYTAELQGYTNG
FG00843.1 	QLIGYDKLPAGQNATVVVMSYSGYDIEDALVLNKASVDRGFGRCQVFRKYTTELQKYPNG
AN0321.1  	ELTKYDQLPAGQNATVAVMSYSGYDIEDALVLNKGSVDRGFGRCQVFRKYVTNMKSYSNG
          	:*  **:*********.*****************.* ************:.:::: *.*.

MG06984.1 	KSEKISGPEIDERTGKP---AEKYAAIDSDGLARVGYRIHSGEVMLRKLTPIDQGSTGIG
NCU01772.1	RRDRVGGIMKDE-DDKP---IVKHAILDADGLAQVGSKVESGQTMIKKETPLDQISTGIA
FG00843.1 	RRERIGDPEMEG-EGKSKRRITKHEALDDDGLAIVGYKVHSGEAMVKKETPLDQTSTGIG
AN0321.1  	TKDILSPPTYEN--DAP---IRKHALLESDGLAAVGEQVNAGEVYINKSTPDQSMSSGMP
          	  : :.    :   . .     *:  :: **** ** ::.:*:. :.* ** :. *:*: 

MG06984.1 	N-DVG-SNDYRETSLSYRVSNPSYIDKVMVSQTEKEHILAKVQTRQTRRPELGDKFSSRH
NCU01772.1	G-DRG-SDDYRDASTNYRISDPAIIDKVMISVNERENKLIKVQTRQTRRPELGDKFSSRH
FG00843.1 	L-DRG-PGEFRDSSVSYRIADPAYIDKVMISQTEKDTTVIKVQTRQTRRPELGDKFSSRH
AN0321.1  	GSDAGRPISYNPTPMTYKLPDPAYIDKVMISATENENQIIKVLTRQTRRPEVGDKFSSRH
          	 :* * . .:. :. .*::.:*: *****:* .*.:  : ** ********:********

MG06984.1 	GQKGVVGIIVEQEDMPFADSGLVPDIIMNPHGFPSRMTVGKLFECLTGKASIVEGNRRYG
NCU01772.1	GQKGVVGIIVNEEDMPFSDKGLRPDIIMNPHGFPSRMTVGKLFECLTGKASVIAGEKDYG
FG00843.1 	GQKGVVGIIVEQEDLPFSDSGLSPDIIMNPHGFPSRMTVGKLLECLTGKASIIHGRPDYG
AN0321.1  	GQKGVTGIIVDQADMPFTDQGINPDIIMNPHGFPSRMTVGKMLELVAGKAGVLSG--QHG
          	*****.****:: *:**:*.*: ******************::* ::***.:: *   :*

MG06984.1 	YGDAFRSHPVEEMGKALIERGFSWEGKDYFTSGITGEPLEAYLFNGPIYYQRLKHMVQDK
NCU01772.1	FGDAFRSHPVESMGQALIDHGFSWEGKDYFTSGITGEPMEAYIYNGPIFYQRLKHMVQDK
FG00843.1 	FGDAFRSHPLEEMSQVLVDHGFSWEGKDYFTSGITGEPLEAYVFNGPIYYQRLKHMVQDK
AN0321.1  	YGTCFSGTPVEQMTQTLIDKGFSYGGKDYLTSGITGEALPFYVFTGPIYYQKLKHMVQDK
          	:* .* . *:*.* :.*:::***: ****:*******.:  *::.***:**:********

MG06984.1 	MHSRAKGPRAILTRQPTEGRSRDGGLRLGEMERDCLIAYGASQLLLERLMLSSDATKIDI
NCU01772.1	MHSRSRGPRAILTRQPTEGRSRDGGLRLGEMERDCLIAYGASQLLLERLMISSDGTELDV
FG00843.1 	MHSRSRGPRAILTRQPTEGRSRDGGLRLGEMERDCLIAYGASQLLLERLMISSDGTEIDI
AN0321.1  	MHSRARGPRATLTRQPTEGRSRDGGLRLGEMERDCLIAYGTSQLLLERLMISSDRHEVDV
          	****::**** *****************************:*********:***  ::*:

MG06984.1 	CQRCGLFGYKGYCQTCKSTKEVTEMTMPYAAKLLVQELISMNVGVRLVLEDEFPHPD--
NCU01772.1	CQECGLFGYKGYCNACKSTRNVTQMTMPYACKLLVQELISMNVGVKLKLEDEFPHPR--
FG00843.1 	CQQCGLFGYKGYCHTCKSTREVTKMTMPYAAKLLVQELISMNVGVRLQMDDEFPHPR--
AN0321.1  	CEQCGFMGYLNWCQRCKSSRSVVKMVIPYAAKLLIQELMSMNVTARLKLEDEFPETKGR
          	*:.**::** .:*: ***::.*.:*.:***.***:***:**** .:* ::****.. .
```
